# Consecutive entosis stages in human substrate-dependent cultured cells

**DOI:** 10.1038/s41598-017-12867-6

**Published:** 2017-10-02

**Authors:** Anastasiia S. Garanina, Olga P. Kisurina-Evgenieva, Maria V. Erokhina, Elena A. Smirnova, Valentina M. Factor, Galina E. Onishchenko

**Affiliations:** 1 Department of Cell Biology and Histology, Faculty of Biology, Lomonosov Moscow State University, Moscow, Russia; 2 Laboratory of Molecular Pharmacology, Center for Cancer Research, National Cancer Institute, National Institutes of Health, Bethesda, MD USA

## Abstract

Entosis, or cell death by invading another cell, is typical for tumor epithelial cells. The formation of cell-in-cell structures is extensively studied in suspension cultures, but remains poorly understood in substrate-dependent cells. Here, we used electron, confocal and time-lapse microscopy in combination with pharmacological inhibition of intracellular components to study the kinetics of entosis using two human substrate-dependent tumor cultures, A431 and MCF7. In total, we identified and characterized five consecutive stages of entosis, which were common for both examined cell lines. We further demonstrated that actin filaments in the entotic as well as invading cells were crucial for entosis. Microtubules and the Golgi apparatus of entotic cells provided membrane expansion required for internalization of the invading cell. Depolymerization of microfilaments and microtubules, and disintegration of the Golgi complex inhibited entosis. We confirmed the presence of adhesive junctions and discovered the formation of desmosomes between the invading and entotic cells. The internalized cell was shown to be degraded due to the lysosomal activation in both cells whereas the disintegration of the Golgi apparatus did not affect the process. Thus, in the substrate-dependent cultures, entosis requires microfilaments, microtubules and the Golgi complex for cell invasion, but not for internalized cell degradation.

## Introduction

Programmed cell death is an integral part of the life of multicellular organism^1,2^. To date many types of cell death have been described in detail. In 2009, the Nomenclature Committee on Cell Death included a new type of nonapoptotic death program triggered by cell-in-cell invasion^3^. The process of active invasion of a live cell into another cell was first described by Overholtzer *et al*. in the matrix-detached mammary epithelial cells and termed “entosis”^4–9^. Later, cell death by invasion has been also discovered in the matrix-attached cells^10,11^. Entosis initiated by invading of a live cell into neighboring cell, also known as cell cannibalism^12^, is frequently found in a wide variety of human tumors mainly of epithelial origin^11,13,14^. In this regard, it is important to characterize the features and mechanisms of entosis to find new targets for chemotherapy.

Previous studies demonstrated that entosis depends on E-cadherin, the cell adhesion protein transducing a contractile force between the actomyosin cytoskeleton and plasma membrane^15^. The actomyosin complex of the invading cell plays an important role during penetration^4,5,16,17^. In addition, the microtubule-based cytoskeleton participates in entosis by regulating cell rigidity^18^. The most commonly observed fate of the internalized cell is degradation via mechanisms controlled by autophagy pathway proteins and lysosomes^4,19^.

Most of the mechanistic studies on entosis were performed on cultured epithelial cells artificially converted to suspension. Here, we aimed to characterize the process of entosis in two substrate-dependent human tumor cell lines, epidermoid carcinoma A431 and mammary adenocarcinoma MCF7. By employing electron, confocal and time-lapse microscopy and pharmacological inhibition of cellular organelles, we identified five consecutive entosis stages and defined the individual roles of the major cellular organelles contributing to invasion and eventual degradation of internalized cells. More specifically, we showed that experimental disassembly of actin filaments by cytochalasin B, microtubules by nocodazole and disorganization of the Golgi apparatus with brefeldin A impeded the course of cell invasion but did not interfere with the degradation of already internalized cells. Our results revealed a previously unrecognized role of outer cell microtubules and the Golgi apparatus during invasion and supported a defining role of lysosomes of inner and outer cells for degradation of internalized cell.

## Results

### Entosis in A431 and MCF7 cells

**Figure 1:**
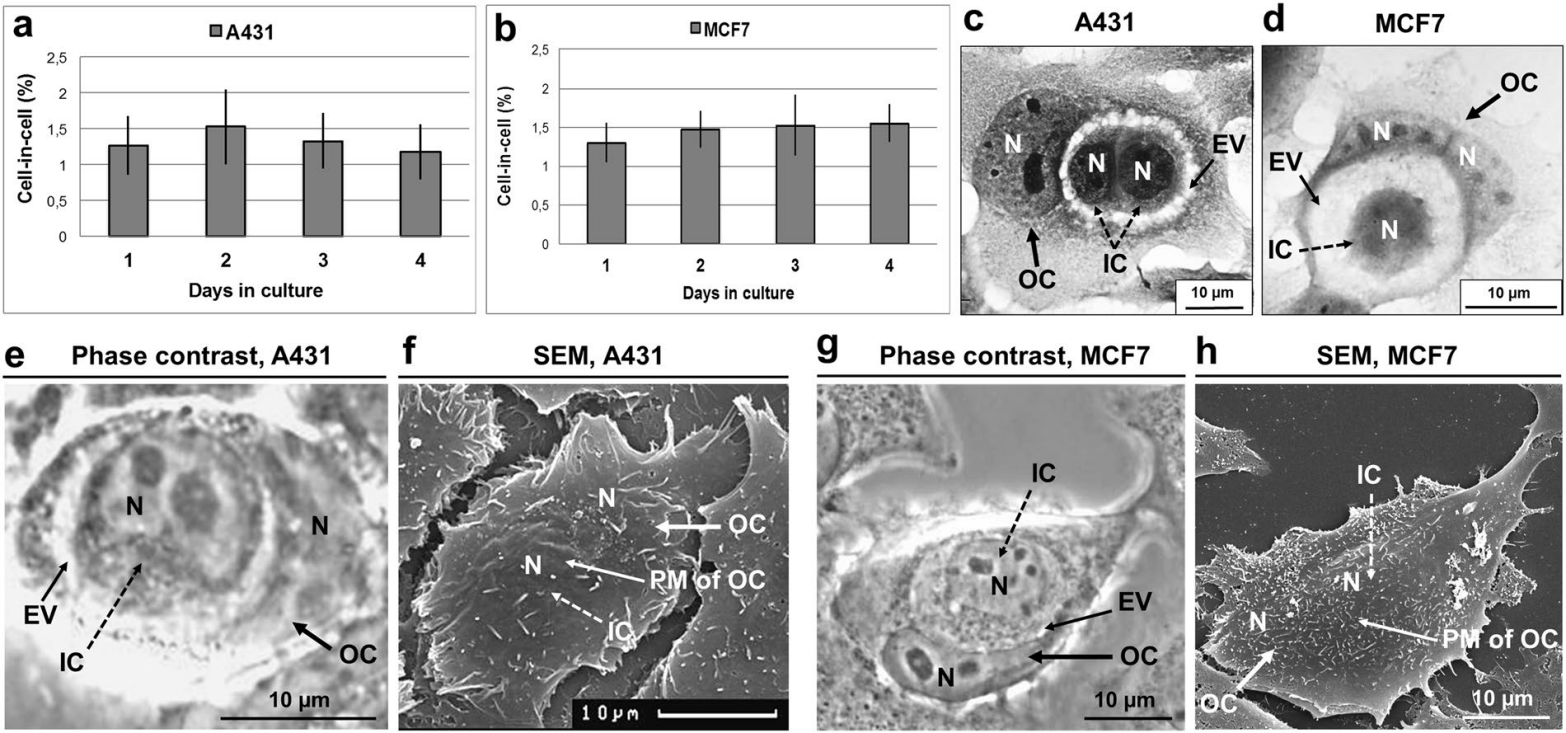
Entosis in A431 and MCF7 cell monolayers. (**a**,**b**) Time-course changes in the frequency of entosis in A431 (**a**) and MCF7 (**b**) cell cultures. Results are shown as means ± SD. n = 1,000 cells were counted per each of three independent experiments. (**c**,**d**) Representative light micrographs of the cell-in-cell structures stained with hematoxylin and eosin demonstrate the morphological changes of inner (IC) and outer (OC) cells. In (**c**), two A431 round-shaped ICs with compact cytoplasm are localized inside the entotic vacuole (EV) of the OC. In (**d**), MCF7 IC with round shape is situated inside the large entotic vacuole of the OC with two nuclei (N). The nuclei of the entotic cells have crescent-like shape and are pushed to the cell periphery. (**e**–**h**) Correlative light-electron microscopy providing evidence of cell localization inside another cell. Representative phase-contrast micrographs of the cell-in-cell structures and scanning electron micrographs of the same entotic cell in A431 (**e**,**f**) and MCF7 (**g**,**h**) cultures. Note that ICs are completely covered by the plasma membranes (PM) of OCs.

A431 cells as well as MCF7 cells undergo entosis at constant rate (1,5–2%) during the first 4 days of culture (Fig. 1a,b). The number of internalized cells could vary from one to four. The internalized (inner) cell had a rounded shape with compact cytoplasm and appeared to be inside a large vacuole (entotic vacuole) (Fig. 1c,d). The nucleus of the entotic (outer) cell acquired a crescent-like shape and was pushed to cell periphery (Fig. 1c–e,g). To confirm that the inner cell was surrounded by the plasma membrane of the entotic cell, we used correlative light-scanning electron microscopy, based on the analysis of the same entotic cells by phase-contrast microscopy followed by scanning electron microscopy. This method demonstrated a complete penetration of one cell into another (Fig. 1f,h).

### Morphological stages of entosis

**Figure 2:**
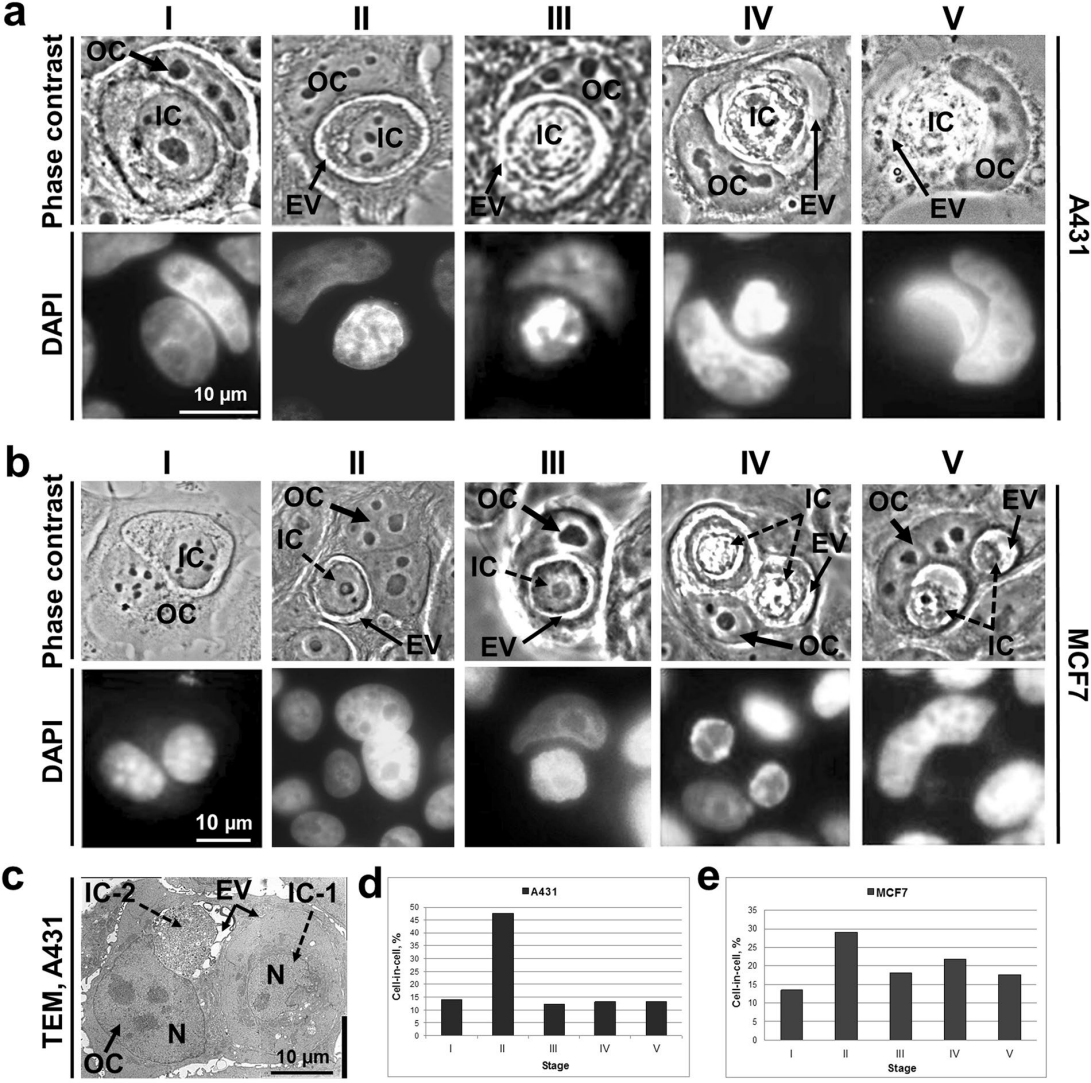
Characterization of entosis stages in A431 and MCF7 cell monolayers. (**a**,**b**) Phase-contrast micrographs (top) and corresponding fluorescence images of DAPI staining (bottom) of entotic cells in A431 (**a**) and MCF7 (**b**) cultures. Note similarities in the consecutive changes in the morphology during five stages of entosis (I–V). IC, inner cell; OC, outer cell; EV, entotic vacuole. (**c**) Transmission electron micrograph (TEM) of two ICs at the different entosis stages (IC-1, stage I; IC-2, stage V) localized inside the same OC. The differences in the morphology of ICs at various entosis stages are visible. (**d,e**) The frequency of cell-in-cell structures expressed as a percentage of total counted cells in A431 (**d**, n = 266) and MCF7 (**e**, n = 193) cultures.

Next we performed a detailed examination of morphological changes of inner cell shape, structure of nucleus, and state of cytoplasm during entosis. In total, we identified five stages of entosis after the cell-in-cell structure formation. The morphology of each stage was very similar both in A431 and MCF7 substrate-dependent cells. The sequence of images shown in Fig. 2 demonstrates that at the first stage, the internalized cell had a rounded shape and retained the size typical for each parental cell in suspension (Fig. 2a-I,b-I and c-IC-1). The plasma membrane of the inner cell was in close contact with entotic vacuole membrane. The nucleus stayed round with a predominance of the diffuse chromatin and several nucleoli. During the second stage, the internalized cell shrank and produced short protrusions extending from the cell body towards entotic vacuole membrane (Fig. 2a-II and b-II). The third stage was characterized by a further decrease in the inner cell size as well as acquisition of irregular shape of both cell and nucleus, and accumulation of cytoplasmic vacuoles in addition to chromatin condensation and less visible nucleoli (Fig. 2a-III, b-III). At the fourth stage, there was a more progressive deformation of cell and nucleus shape, increased accumulation of cytoplasmic vacuoles, and disappearance of nucleoli (Fig. 2a-IV,b-IV). At the final fifth stage, the entotic cell contained only a remnant of inner cell (Fig. 2a-V,b-V and c-IC-2) with a tightly condensed nuclear chromatin and intensive vacuolization of cytoplasm. In some cases, there remained nuclear remnants of the inner cell at the last stage of entosis. We quantified the incidence of different entosis stages. The most frequent was the second stage in both types of cultured cells (Fig. 2d,e). All other stages were observed at approximately the same frequency.

**Figure 3:**
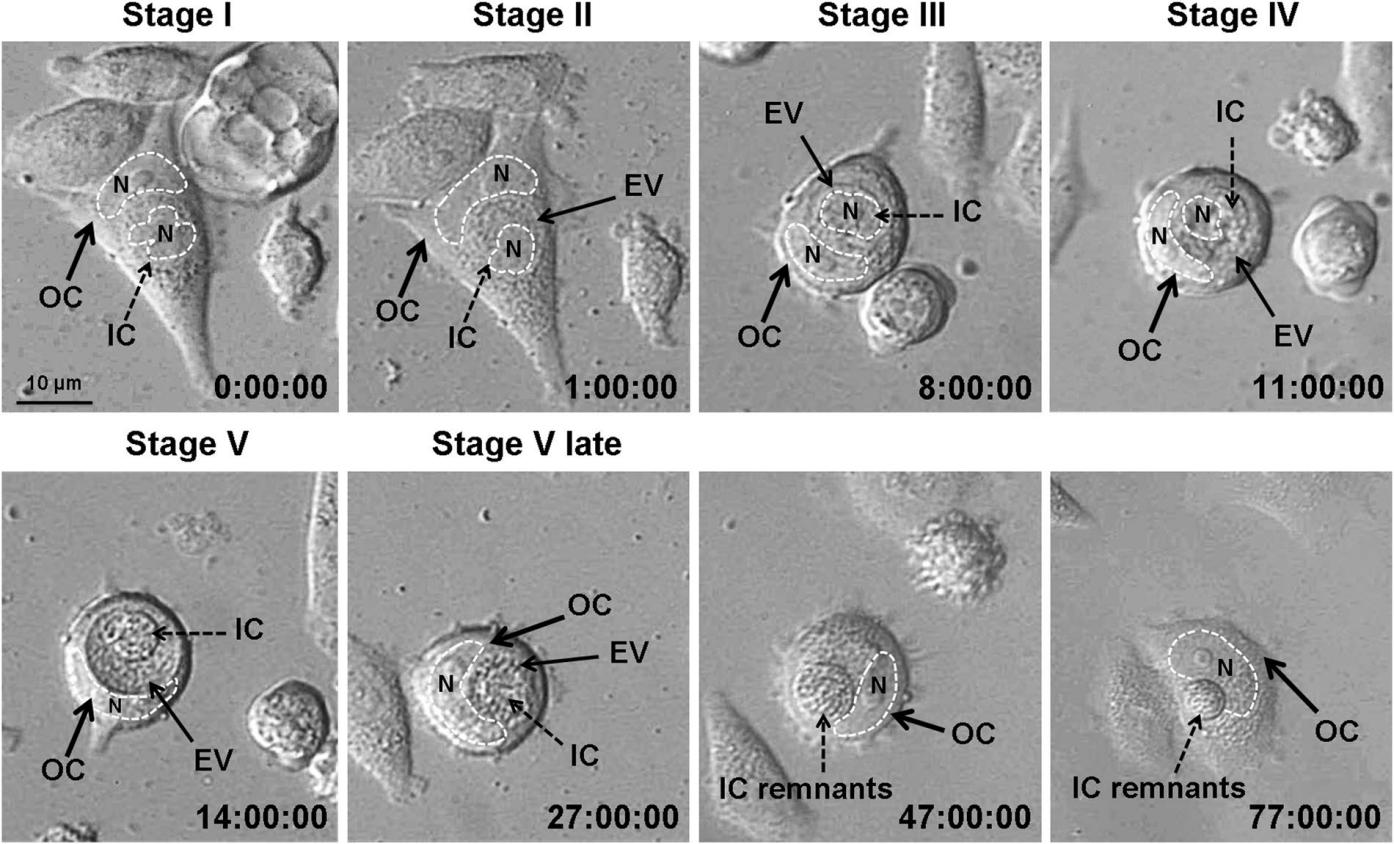
Representative time-lapse imaging of entosis in a MCF7 cell monolayer. Shown is the full sequence of events from cell penetration into another cell and up to its degradation. IC, inner cell; OC, outer cell; EV, entotic vacuole. Nuclei (N) are encircled by the dashed lines. In 9 out of 13 examined cells, the inner cells were degraded at the end of entosis, while in the remaining 4 cells the inner cell exited the entotic vacuole.

To corroborate the entosis staging based on the sequential analysis of the individual images, we then employed time-lapse microscopy. Data are shown for MCF7 cells (Fig. 3). Images obtained for A431 cells demonstrated the same results, but fewer cells were analyzed because of their high motility. Altogether, we traced thirteen MCF7 entotic cells during three independent experiments. In nine out of thirteen cells, the inner cell was degraded at the end of entosis, while in the remaining four cells the inner cell exited the entotic vacuole. Time-lapse imaging was consistent with the described sequence of the morphological events characteristic for stages I–V of entosis. It should be noted that the size of entotic vacuole was reduced at the fifth entosis stage. Remnants of the internalized cell could be found at this stage. The entotic cell also changed during entosis. First, it was flat and developed obvious lamellae (stages I–II), then it shrank and acquired a rounded shape (stages III–V), and finally the entotic cell became flattened again while the inner cell debris was still visible (after stage V).

Based on the time-lapse microscopy, the approximate duration of each of the 5 entosis stages was estimated as 1, 7, 3, 3 and > 24 hours, respectively.

### Cell–in-cell structure requires adhesive junctions between cells

**Figure 4:**
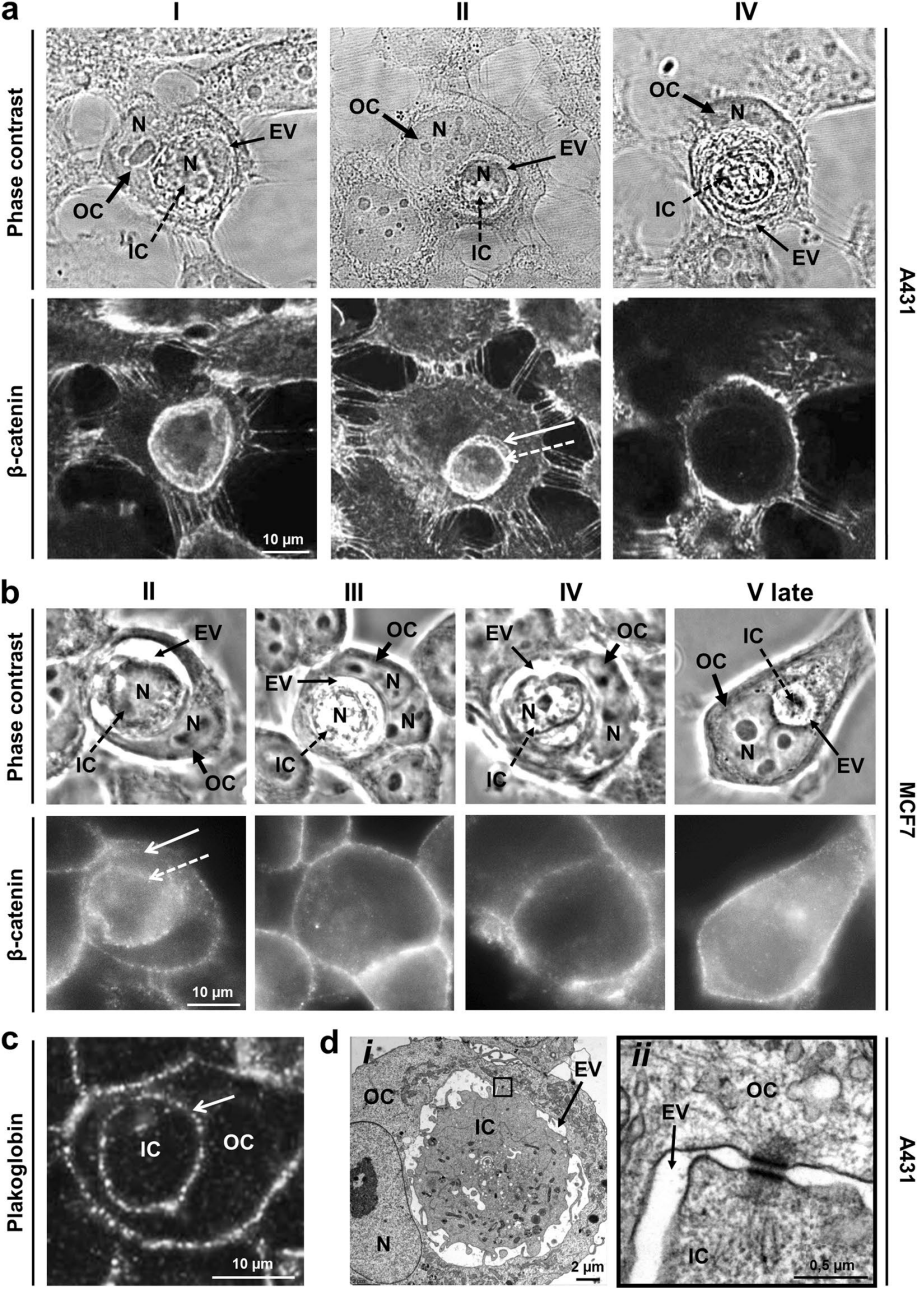
Formation of intercellular junctions between inner and outer cells. (**a**,**b**, top) Representative phase-contrast micrographs. (**a**,**b**, bottom) Confocal laser scanning (**a**) and fluorescence (**b**) images of staining with anti-β-catenin antibodies of entotic cells in A431 (**a**) and MCF7 (**b**) cultures show disappearance of adhesive junctions between the inner (IC) and outer (OC) cells during entosis. Note a single thick β-catenin-positive band between the membranes of IC and entotic vacuole (EV) at stage I, two bands of β-catenin along the periphery of IC and EV at stage II, and absence of β-catenin staining in the EV area at the later entosis stages. N, nucleus. Dashed arrow, IC plasma membrane; arrow, EV membrane. (**c**,**d**) Desmosomes formed between IC and OC are disassembled during entosis. (**c**) Confocal laser scanning micrograph of entotic cell stained with anti-plakoglobin antibodies (stage I) demonstrates the presence of numerous desmosomes between the IC and OC over the whole periphery of IC. (**d**) Transmission electron micrographs of desmosomes between IC and OC (stage II). Individual desmosomes are located at the terminal ends of the plasma membrane protrusions. Panel (*ii*) is a magnified view of the boxed area in panel *i*.

It was reported that initiation of entosis in suspension cells requires formation of adhesive junctions with E-cadherin and β-catenin^4^. Similarly, the dense β-catenin plaques were detected in A431 and MCF7 adherent cells at the first stage of entosis. Morphologically, they appeared as single thick bands between the membranes of internalized cell and the entotic vacuole (Fig. 4a-I). The second entosis stage was defined by the presence of two β-catenin bands located along the periphery of inner cell and entotic vacuole (Fig. 4a-II ad b-II). At the later stages, β-catenin staining retained only in the outer cell plasma membrane (Fig. 4a-IV and b-III–V late).

Specialized junctions such as desmosomes are inherent for epithelial cells^20^. Therefore, we next examined the localization of desmosomes at different entosis stages in A431 cell culture using antibodies against desmosome protein plakoglobin^21^. During the first stage, plakoglobin plaques were distributed over the periphery of internalized cell (Fig. 4c). At the second stage, some desmosomes were located at the terminal ends of the plasma membrane protrusions. The plakoglobin immunofluorescence staining was no longer detected at the later stages. Transmission electron microscopy analysis confirmed the presence of desmosomes between the entotic and internalized cells (Fig. 4d). This may suggest that both cadherins and specialized adhesive junctions participated in entosis in substrate-dependent cells.

**Figure 5:**
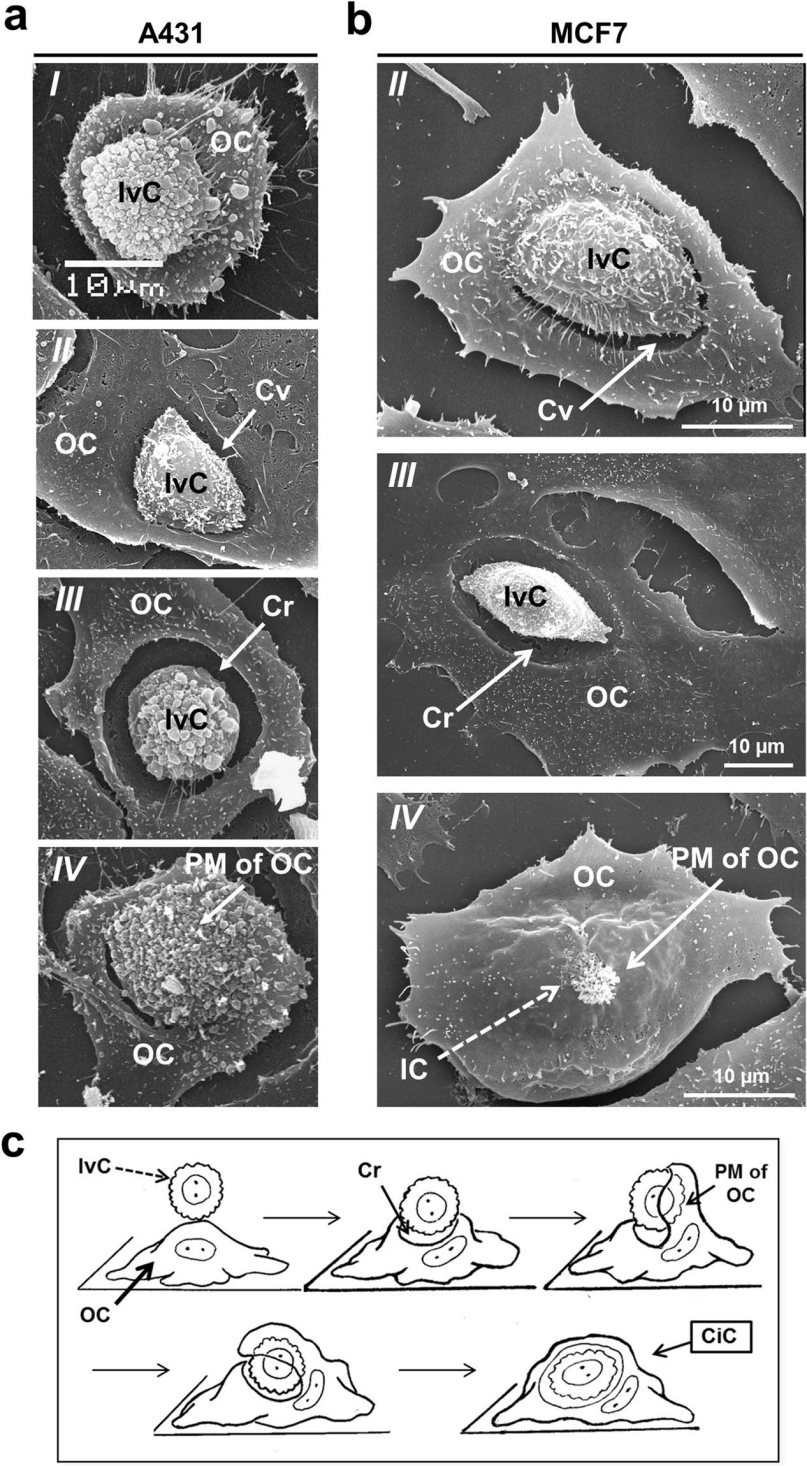
Formation of cell-in-cell structures in A431 and MCF7 cell monolayers. (**a**,**b**) Representative scanning electron micrographs of the invading cells in A431 (**a**) and MCF7 (**b**) cell monolayers. Panel *I* shows attachment of the invading cell (IvC) to the attached cell; panels *II* show formation of a cavity (Cv); panels *III* show formation of a deep crater (Cr) in the outer cell (OC) plasma membrane under pressure of IvC; and panels *IV* show a flattened membrane protrusion formation by entotic cell covering the IvC. (**c**) The scheme of the events during cell-in-cell invasion. CiC, cell-in-cell; IC, inner cell; PM of OC, plasma membrane of outer cell.

Previous results demonstrated the activity of the inner cell during invasion in suspension-cultured cells^4,15^. Our studies, using series of scanning electron microscopy images at the different stages of cell internalization (Fig. 5a,b), showed that invading cells took part in entosis, and that entotic cells changed their morphology during this process. First, the invading cell attached to a potential entotic cell causing a deformation of its plasma membrane and a subsequent development of a cavity under the invading cell. Then, the adherent entotic cell enclosed the invading cell with a flat plasma membrane protrusion, designated “flattened membrane protrusion” (Fig. 5c).

### Cell internalization requires intact actin cytoskeleton

**Figure 6:**
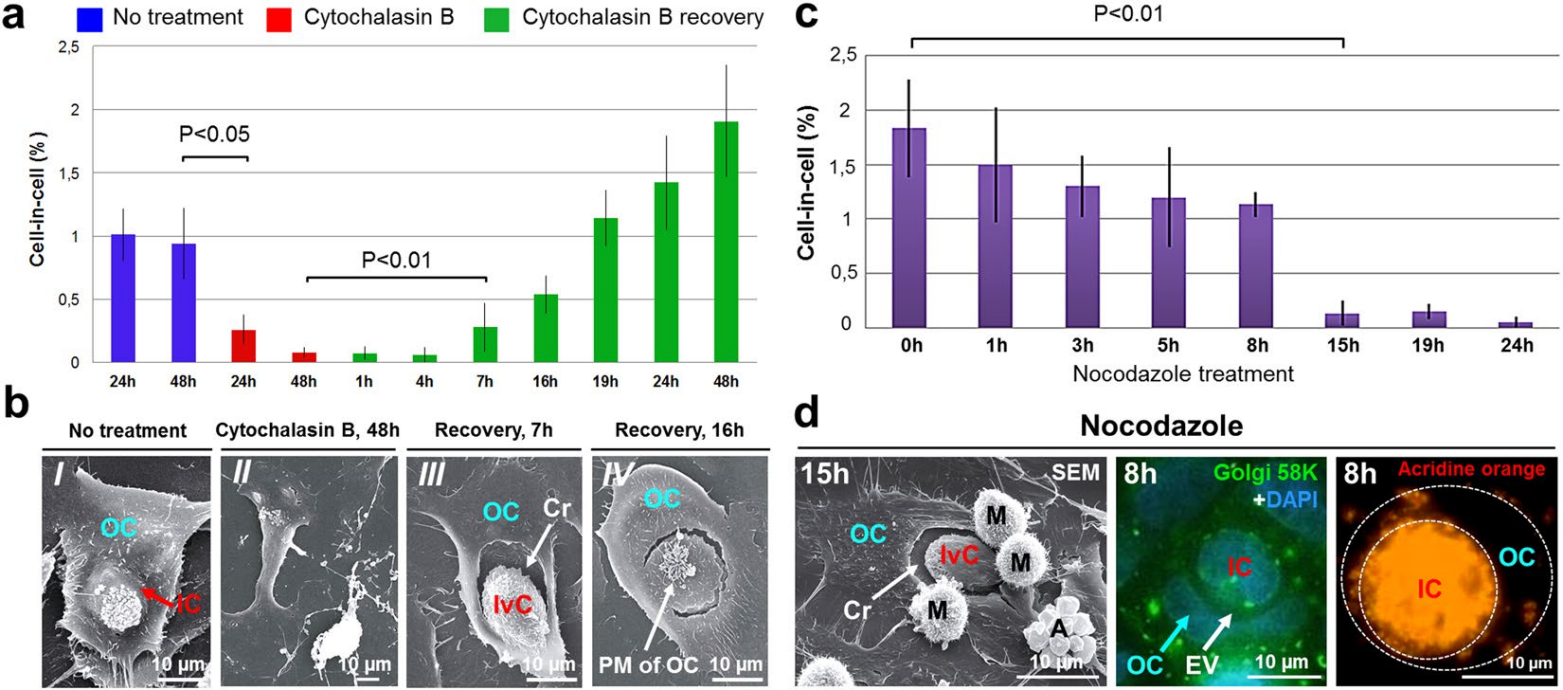
Disorganization of the actin cytoskeleton and microtubules inhibits entosis. (**a**) Time-course changes in the frequency of entosis in A431 cells after cytochalasin B treatment and recovery. Disorganization of actin filaments caused by cytochalasin B results in a significant inhibition of entosis. Recovery after the drug exposure leads to a progressive increase in cell-in-cell structures. Results are shown as means ± SD. n = 1,000 cells were counted per each of three independent experiments. (**b**) Scanning electron micrographs of entotic cells (*I*), cells treated with cytochalasin B for 48 h (*II*), crater (Cr) formation in the entotic cell by the invading cell (IvC) 7 h after cytochalasin B recovery (*III*), and a flattened membrane protrusion formation by entotic cell covering the IvC 16 h after cytochalasin B recovery (*IV*). (**c**) Time-course changes in the frequency of entosis after nocodazole treatment. Microtubule depolymerization leads to a significant reduction in the number of entotic cells. Results are shown as means ± SD. n = 1,000 cells were counted per each of three independent experiments. (**d**) Left panel, scanning electron micrograph (SEM) of the “IvC” 15 h after nocodazole treatment. Note, that “IvC” forms a Cr in the entotic cell, which doesn’t form a flattened membrane protrusion. Middle and right panels, fluorescence staining with anti-p58K antibodies and DAPI (middle) and acridine orange (right) demonstrate localization of the Golgi apparatus (green) and lysosomes (orange) in inner (IC) and outer (OC) cells 8 h after nocodazole treatment. Middle panel shows disintegration of the Golgi apparatus. Right panel shows that lysosome-mediated degradation of the IC is not inhibited by nocodazole treatment. A, apoptotic cell; EV, entotic vacuole; M, mitotic cell; PM of OC, plasma membrane of outer cell.

It was previously shown that cell invasion depends on actin polymerization in invading cell^4^. We suggested that the actin cytoskeleton of entotic cell should also participate in this process. Since actin filaments are required for a flattened membrane protrusion formation^22,23^, we assumed that they play the same role during a flattened membrane protrusion formation by entotic cell (Fig. 5c). To confirm the critical role of actin organization during entosis, the A431 cells were cultured for 48 h in the presence of cytochalasin B, a known inhibitor of actin polymerization^24,25^. As expected, cytochalasin B treatment significantly inhibited entosis starting from 8 h (3-fold reduction, *P* < 0.05). By 48 h, the rate of entosis was decreased by about 10-fold (*P* < 0.05) (Fig. 6a). This was paralleled by profound changes in cell morphology as judged by scanning electron microscopy. All cells acquired an irregular shape, became more flattened, lost plasma membrane microvilli, and more importantly, did not form surface “craters” (Fig. 6b-II). No early stages of entosis were observed after 8 h of incubation with cytochalasin B, and only completely degraded internalized cells were found inside the entotic cells after 48 h.

The inhibitory effect of cytochalasin B was reversible. The number of internalized cells progressively increased after the drug removal. The initial stages of entosis were detected after 4–7 h of recovery, a flattened membrane protrusions after 16 h, and a complete cell-in-cell formation was found after 24 h (Fig. 6b-III and b-IV). The frequency of entotic cells reached the basal level by 24 h, and exceeded it after 48 h. Thus, disorganization of actin cytoskeleton inhibited entosis in the substrate-dependent cultured cells by preventing the initiation stage of cell-in-cell structure formation and allowing synchronization of the initiation of entosis.

### Entosis requires intact microtubules

Microtubules regulate cell rigidity that is important for cell invasion during entosis^18^. To test the contribution of microtubule dynamics into temporal and spatial control of entosis in A431 cells, we used nocodazole, a drug affecting microtubule assembly. Nocodazole treatment resulted in a complete disassembly of microtubules starting from 30 min exposure. This was paralleled by a steady decline and finally a complete loss of entotic cells by 24 h (Fig. 6с). In particular, nocodazole affected the early stages of entosis, which disappeared after 8 h of treatment. Notably, scanning electron microscopy analysis revealed that in nocodazole-treated cultures, the invading cell was still capable of forming cavity in the entotic cell (Fig. 6d), but the outer cell didn’t form a flattened membrane protrusion. These data showed that microtubules, together with actin filaments, were involved in the process of cell invasion.

### The intact Golgi apparatus is required for active cell invasion but not for degrading the internalized cell

The Golgi apparatus, the sole source of lysosomes^26,27^, is shown to participate in vesicular transport likely contributing to plasma membrane growth^28,29^ and a flattened membrane protrusion formation.

**Figure 7:**
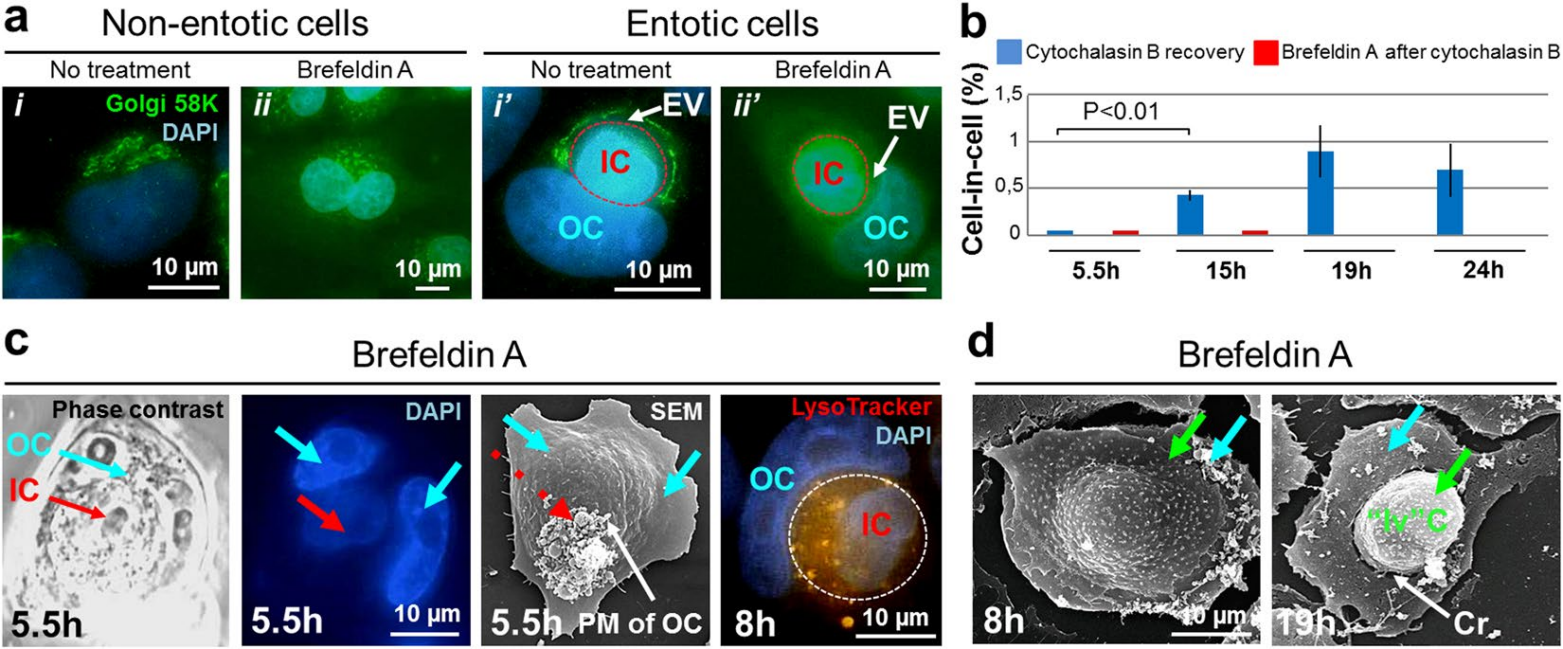
Disassembly of the Golgi apparatus inhibits entosis in A431 cell monolayer. (**a**) Fluorescence micrographs of cells stained with anti-p58K antibodies and DAPI demonstrate localization of the Golgi apparatus in untreated *(i, i*’*)* and brefeldin A-treated *(ii, ii’)* non-entotic and entotic cells. *i*, Golgi is located near the nucleus; *i’*, redistribution of the Golgi apparatus during entosis: Golgi is localized around the entotic vacuole (EV) in outer cell (OC) (stage II). Panels *ii, ii’* show disassembly of the Golgi apparatus in non-entotic and entotic cells after brefeldin A treatment. (**b**) Time-course changes in the frequency of entosis: blue column, 48 h incubation with cytochalasin B followed by a recovery for 5.5, 15, 19 and 24 h; red column, 48 h incubation with cytochalasin B followed by a recovery for 15 min and treatment with brefeldin A for 5.5, 15, 19 and 24 h. Note a gradual increase in cell-in-cell structures after cytochalasin B recovery whereas an additional brefeldin A treatment caused a complete inhibition of entosis. Results are shown as means ± SD. n = 1,000 cells were counted per each of three independent experiments. (**c**) Correlative light and electron microscopy of cell-in-cell structure 5.5 h after brefeldin A treatment. Shown are representative phase-contrast micrograph, DAPI staining, and scanning electron micrograph (SEM) of the same cell-in-cell structure. The inner cell (IC) is covered by the plasma membrane of OC. Red arrow, IC; blue arrows, two nuclei of the entotic cell; dashed red arrow, protuberances of OC plasma membrane. Right panel shows the pattern of lysosome staining with LysoTracker (orange) of IC and OC 8 h after brefeldin A treatment. PM of OC, plasma membrane of outer cell. (**d**) Scanning electron micrographs of cells 8 h (left) and 19 h (right) after brefeldin A treatment. Green arrows point to the cell spreading over the apical surface of the substrate-attached cell (left) and to the round-shaped cell located at the crater-like (Cr) deformation of the substrate-attached cell plasma membrane (right). Note, that the plasma membrane of substrate-attached cell doesn’t cover such “invading” cell (“Iv”C). Blue arrows, substrate-attached cells.

To address a role of the Golgi apparatus during entosis, we first performed immunofluorescence staining of A431 cells with anti-Golgi 58 K protein antibody. The results showed that the Golgi apparatus of internalized cell maintained the compact structure and perinuclear localization typical for this culture during the first I-II and occasionally stage III of entosis. In contrast, in the outer cell, Golgi apparatus was located around the entotic vacuole throughout I–IV stages of entosis (Fig. 7a-*i*’) and in perinuclear area at stage V.

To further address the Golgi contribution to entosis, we used brefeldin A (Fig. 7a-*ii* and a-*ii*’), a protein transport inhibitor causing disassembly of the Golgi apparatus^30,31^. To synchronize the start of entosis, the A431 cells were first exposed to cytochalasin B for 48 h, and after 15 min of recovery were treated with brefeldin A.

Quantitative analysis of entosis frequency showed that brefeldin A caused a significant reduction of cell-in-cell formation as compared to control cells treated with cytochalasin B alone (and washed after it) (Fig. 7b). Only occasional entotic cells were found upon brefeldin A treatment (Fig. 7c). Scanning electron microscopy of brefeldin A-treated cells revealed the formation of two-cell structures in which the top cell was attached and spread over the apical surface of the substrate-attached cell (cell over cell spreading) (Fig. 7d, left) or the top rounded cell was located at the crater-like deformation of the plasma membrane of the bottom spread cell (Fig. 7d, right). Interestingly, the spread cell didn’t form a flattened membrane protrusion covering the top rounded cell.

However, brefeldin A did not affect the inner cell degradation (Fig. 7c), suggesting that the latter was executed by the lysosomes formed before treatment.

It is worth noting that in the experiment in which nocodazole treatment caused disintegration of the Golgi apparatus^32^, the degradation of internalized cell was not inhibited and continued after 8 h and longer exposure to drug (Fig. 6d).

### Lysosome activation during entosis

In suspension cultures, internalized cells undergo lysosome-mediated degradation during entosis^4^. To examine the distribution of the low pH vesicular compartment in the substrate-dependent A431 and MCF7 cells, we performed vital fluorescence staining with acridine orange used as a lysosomal dye^33^. The results showed that lysosomes of the inner cell were aggregated in the perinuclear area during entosis stage I (See Supplementary Fig. S1). During stage II, the lysosomes were found at the cell periphery, spread evenly over cytoplasm at stage III, and fused at stage IV. The number of lysosomes progressively increased through stages I–V. The lysosome re-distribution in the internalized cell occurred in parallel with cytoplasm acidification, and was followed by extensive acidification of the entire entotic vacuole by stage V.

The outer cell retained a small number of lysosomes at all entosis stages. The lysosomes were located around the entotic vacuole during stages I–II, and near the cell nucleus at the later stages. The same changes in the acidic compartments during entosis were found in MCF7 cells.

### The mitochondria of internalized cells retain functional activity during early entosis stages

The low pH inside lysosomes is maintained by the ATP-dependent proton pumps^34^. We assumed that energy generated by mitochondria is required for degrading the internalized cell. To monitor the membrane potential of mitochondria, the A431 cells were vital stained with the potential-dependent dye rhodamine 123. The result showed that the mitochondria of the inner cell were functional and capable of maintaining the membrane potential until stage III (See Supplementary Fig. S1). At stages IV–V, the mitochondrial activity was reduced, and rhodamine 123 staining was no longer detected. The change in the internalized cell mitochondria state was confirmed by ultrastructural analysis with transmission electron microscopy. The mitochondrial matrix gained a more dense consistency, which is typical for mitochondria with low membrane potential^35^. The mitochondria of the outer cell were located mainly around the entotic vacuole at all entosis stages. Their ultrastructure was characteristic of normally functioning cells.

### Entosis is a non-apoptotic process

The death of the internalized cell during entosis usually is not associated with apoptotic mechanisms and occurs by lysosome-mediated degradation^4,19,36^. However, it was reported that in some cases a small part of the internalized cells dies by apoptosis pathway^10^. Of note, caspase-3 activation inside the internalized cell was found during another type of cell cannibalism, emperipolesis, when the A431 cell was an outer cell^37^. To investigate whether the inner cell dies by apoptotic pathway during entosis in the human epidermoid cell line, we stained A431 cells with cytochrome *c* and active caspase-3 antibodies as well as with vital dye 2′,7′-dichlorofluorescein diacetate (DCFH-DA), which detects the reactive oxygen species (ROS) in cells. Diffuse staining of the cell cytoplasm demonstrating cytochrome *c* release from mitochondria, caspase-3 activation and accumulation of ROS were observed during apoptosis of mononuclear cells. However, none of these three types of staining was detected during entosis (See Supplementary Fig. S2). Based on these findings, we conclude that entosis of A431 cells is non-apoptotic cell death.

## Discussion

In this study, we provide a detailed description of the kinetics of entosis in two types of substrate-dependent cultured cells, A431 and MCF7. By applying confocal and electron microscopy in combination with pharmacological inhibition of the intracellular organelles, we define both common and unique features differentiating the process of entosis between adherent and suspension cultured cells.

In agreement with recent studies on suspension cultures^4,15^, we demonstrate a direct link between the cells involved in entosis via formation of adhesive junctions in A431 and MCF7 substrate-dependent cultures. Furthermore, we discovered the development of specialized adhesive junctions, such as desmosomes, in A431 cells. These findings suggest that both cadherins and specialized adhesive junctions, such as desmosomes, common in MCF7 cells^38^, may play a role during entosis in the substrate-dependent cultured cells. Although the contribution of adherent molecules E-cadherin and α-/β-catenin to entosis is shown^36^, the assembly of desmosomes and their functional role during entosis requires further investigation.

The fact that entosis is initiated by cell detachment from matrix^4–9^ implies that adhesive junctions found between the outer and inner cells are formed *de novo* during entosis. These data confirm the important role of intercellular contacts in the process of cell invasion. Moreover, this type of interaction disappears upon transformation of the outer cell plasma membrane into the membrane of entotic vacuole, which includes LAMP1^4^ and autophagy proteins^39–41^ suggesting that strong adhesion is required for the retention of the invading cell inside the developing entotic vacuole. The release of the internalized cell found in some cases^14,42^ may be likely explained by the absence of such junctions.

Another defining feature of entosis in adherent cells was formation of a flattened membrane protrusion by outer cells, which encircled the invading cells. Microtubules and the Golgi apparatus of entotic cell provided the increase of the outer cell surface area required for a flattened membrane protrusion formation. Taken together, our morphological and inhibitor-based studies may suggest that the outer cell in adherent cultures is taking a more active role in the cell-in-cell structure formation while its role during entosis in cells cultured in suspension seems less significant. However, more work is needed to address this possibility using fluorescently labeled cells and/or loss-of-function studies specific to outer cells.

In our study, we also showed that microtubule depolymerization by nocodazole led to inhibition of entosis. It’s known that the organelle and vesicular transport inside the cell are provided by microtubules^43–45^. We suggest that in the substrate-dependent cultures, the role of microtubules during entosis extends beyond regulation of cell rigidity^18^ to include delivery of the additional membrane vesicles to the outer cell plasma membrane. The latter is important for increasing the entotic cell surface needed for crater and a flattened membrane protrusion formation during cell invasion.

It was previously described that mobilization of the actin cytoskeleton in the invading cells is required for cell-in-cell structure formation in suspension culture^15^. We confirmed and extended these findings. We showed that intact actin filaments in both outer and inner cells contributed to cell-in-cell invasion in the substrate-dependent cells as judged by the experiments in which cytochalasin B treatment resulted in a significant reduction of entotic index. We also established that the main role of the actin cytoskeleton in outer cell was creation of the crater and a flattened membrane protrusion surrounding the invading cell. The impact of the outer cell cytoskeleton after the cell-in-cell structure formation seems to be less significant. Of note, the disassembly of actin filaments by 48 h treatment with cytochalasin B followed by a period of recovery allowed synchronization of the initiation of entosis. We suggest that this approach may serve as a helpful tool to advance research of the consecutive entosis stages.

The experiments with brefeldin A treatment revealed an important contribution of the Golgi apparatus to entosis. First, the Golgi apparatus participated in formation of the additional membranes required for the outer cell surface increase during cell internalization^28,29^. Second, the Golgi apparatus mediated lysosome formation and maturation^26,27^. At the early stages of entosis, the presence of lysosomes in the outer cell was required for the interaction with the entotic vacuole in order to modify its membrane and to release hydrolases inside the vacuole for providing the inner cell degradation. However, to date, a complete picture of the events leading to the transformation of the outer cell plasma membrane into the entotic vacuole membrane is still missing. Autophagy proteins (e.g. Atg5, Atg7, Vps34 and LC3) are shown to modify the entotic cell plasma membrane^40,41^ allowing fusion with outer cell lysosomes. LAMP1, an integral protein of the lysosome membrane, is inserted into the membrane of entotic vacuole, thereby protecting the outer cell from the lysosomal enzymes^46^. However, it is still unknown what signal(s) is required for the membrane transformation. To elucidate the mechanism underlying the internalized cell degradation and the role of lysosomes and autophagy in this process, it is necessary to investigate when the exchange of plasma membrane proteins (e.g. E-cadherin and β-catenin) with the autophagy and lysosome proteins takes place and when lysosomes of the entotic cell begin to fuse with the entotic vacuole. The inner cell degradation after Golgi disintegration caused by nocodazole treatment^47,48^ could be executed by the existing lysosomes.

Based on the morphological changes of internalized cells and time-lapse imaging, we divided entosis into five consecutive stages. During the first stage, the outer and inner cells establish close relationships with each other via formation of adhesive and specialized junctions such as desmosomes along with reconfiguration of the outer cell, leading to the organelle repositioning at the periphery of entotic vacuole. The second stage is most likely characterized by the primary modification of the membrane of the entotic vacuole by autophagy proteins, and activation of acid vesicular organelles in the invading cell. During the third stage, the membrane of the entotic vacuole is modified once again by the membrane proteins of lysosomes. At this stage, the first signs of degradation of the internalized cell are detected. The fourth stage is characterized by lysosome fusion inside the internalized cell. The key features of the fifth stage include strong acidification of the entotic vacuole and relocation of organelles (the Golgi complex and microtubules) of the entotic cell to the initial position. Similar findings were described in an immortalized keratinocyte cell line HaCaT^49^ suggesting that the described sequence of events is common for entosis in the substrate-dependent cells. The fate of the internalized cell’s undigested material awaits further investigation.

Our data confirmed the lysosome-mediated degradation of inner cell during entosis in substrate-dependent A431 and MCF7 cultures and revealed no evidence of apoptosis in the human epidermoid carcinoma cell line.

In conclusion, our study provides new insights into the process of entosis in substrate-dependent tumor cells. The description of the consecutive entosis stages and a simple method of synchronizing the process of cell-in-cell invasion may advance the work towards understanding the molecular and cell biological aspects of entosis and its role in cancer.

## Materials and Methods

### Cell Culture

A431 human epidermoid carcinoma cells and MCF7 human adenocarcinoma cells (Institute of Cytology, Russian Academy of Sciences (INC RAS)) were maintained in Dulbecco modified Eagle’s medium (DMEM) (Paneco) supplemented with 10% Fetal Bovine Serum (“PAA Laboratories”, Austria), 2 mM L-glutamine, and 80 μg/ml of gentamycin. Cells were seeded on glass coverslips at a concentration 200 000 cells per ml and cultured at 37 °C in a humidified incubator supplied with 5% CO_2._ The number of cell-in-cell structures was counted per at least 1,000 cells from three independent experiments and expressed as percent. The number of entotic cells at each entosis stage was counted per all detected cell-in-cell structures and expressed as percent.

### Pharmacological inhibition of actin filaments, microtubules and Golgi complex

Cytochalasin B (Sigma), nocodazole (Sigma) and brefeldin A (Sigma) were diluted in DMSO at a final concentration in culture medium at 5 µg/ml. Cells were exposed to cytochalasin B and nocodazole 2 days after plating and cultured in the presence of cytochalasin B for 1–48 h, and in nocodazole for 1–24 h. Thereafter, cells treated with cytochalasin B, were washed with culture medium three times for 5 min and cultured for the indicated time. Cells grown in the presence of cytochalasin B for 48 h, were subsequently treated with brefeldin A after 15 min of recovery.

### Vital observations

To detect the acid vesicular compartment and ROS, unfixed cells were stained for 30 min with acridine orange (Sigma) and 2′,7′-Dichlorofluorescein diacetate (DCFH-DA) (BioChemika), respectively. Mitochondria were visualized by staining with rhodamine 123 (Sigma). Acridine orange and rhodamine 123 were used at concentration of 1 μg/ml. DCFH-DA was used at concentration of 10 μM.

### Light and confocal microscopy

For morphological analysis, cells were fixed in Bouin solution for 30 min and stained with hematoxylin-eosin (H&E). For immunofluorescence analysis, cells were fixed in 4% formaldehyde (Sigma) for 20 min, permeabilized with 0.2% Triton X-100 in PBS for 7 min and incubated in PBS supplemented with 1% BSA for 1 h. The primary antibodies were mouse monoclonal anti-Golgi 58 K protein/formiminotransferase cyclodeaminase (FTCD) (Sigma, 1:50), mouse monoclonal anti-plakoglobin (catenin γ) antibody (Sigma, 1:100), mouse monoclonal anti-α-tubulin antibody (Sigma, 1:1000), rabbit polyclonal anti-γ-tubulin antibody (Sigma, 1:1000), rabbit anti-β-catenin antibody (Sigma, 1:1000), rabbit monoclonal anti-caspase-3 antibody (Sigma, 1:100), and sheep polyclonal anti-cytochrome *c* antibody (Sigma, 1:100). Alexa Fluor 488 goat anti-mouse IgG (H + L) (Invitrogen, 1:800), Alexa Fluor 568 donkey anti-rabbit IgG (Invitrogen, 1:800) and Alexa Fluor 546 donkey anti-sheep IgG (Invitrogen, 1:800) were used as secondary antibodies. DNA was stained with DAPI (Sigma) for 10 min.

The acid vesicular compartment was also detected by vital staining with LysoTracker Red DND-99 (Invitrogen) for 30 min. Thereafter, cells were washed with PBS and fixed with 4% formaldehyde. F-actin was detected with TRITC-phalloidin (Sigma).

Images were taken with a Leica light microscope, an Axiovert 200 M fluorescence microscope (“Carl Zeiss Inc.”), a Leica TCS SPE and a Leica TCS SP2 laser scanning confocal microscopes, and processed using the NIH ImageJ software.

### Transmission electron microscopy (TEM)

Cells were fixed in a mixture of 2.5% glutaraldehyde and 2% formalin in 0.1 M PBS buffer (pH 7.4) for 1 h and post-fixed in 1% osmium tetroxide (OsO_4_) for 1 h in the dark. After samples were washed in 0.1 M PBS (10 min) and distilled water (2 × 10 min), they were dehydrated in a graded series of ethanol solutions, including 50% (4 × 10 min, 4 °C in the dark), 60% (2 × 20 min, 4 °C), 70% with 1.5% uranyl acetate (15 min, 4 °C followed by overnight incubation at 4 °C), 80% (15 min, 4 °C), 96% (15 min, room temperature), 100% (3 × 30 min, room temperature), acetone (3 × 30 min), and in mixtures of acetone:Epon resin (Epon 812, Fluka Chemikals) at a ratio of 3:1 (60 min); 1:1 (60 min) and 1:3 (overnight). Finally, the samples were incubated in a clean epoxy resin (6 h) and embedded in Epon resin, which was allowed to polymerize for 24 h at 37 °C and 48 h at 60 °C^50^. Ultrathin sections (70 nm) were cut with LKB II ultramicrotome using a diamond knife and placed on electron microscopy one-slot grids coated with Formvar film. The samples were counterstained with 1.5% uranyl acetate solution in water (20 min in a humid chamber in the dark) and lead citrate (5 min in the presence of sodium hydroxide^51^), and examined in JEM-1011 (100 kV) transmission electron microscope connected to a GATAN ES500W camera driven by Digital Micrograph software and transmission electron microscope JEM-100B (JEOL).

### Scanning electron microscopy (SEM)

Cells were fixed in 2.5% glutaraldehyde with 2% formalin in 0.1 M PBS buffer (pH 7.4, 1 h), post-fixed in 1% osmium tetroxide in distilled water (1 h in the dark), washed in PBS (2 × 5 min), and dehydrated in a standard series of ethanol solutions (30%, 50%, 70%, 82%, 96%, 100% 2 × 5 min each), followed by 96% ethanol: acetone solutions (mixed at 3:1, 1:1, and 1:3 ratio, 2 × 5 min each), and finally in acetone (10 min). The samples were dried at a critical point in CO_2_ (t = 31 °C, р = 73.8 bar) and coated with the gold-palladium by sputtering^52^. The identified cells of interest were viewed under SCAN JSM-6380LA (JEOL) microscope.

### Correlative light-electron microscopy (CLEM)

Cells were fixed in 2.5% glutaraldehyde with 2% formalin in 0.1 M PBS buffer (pH 7.4) for 1 h. DNA was stained with DAPI for 10 min. After phase-contrast and fluorescent images of cell-in-cell structures were taken (by Axiovert 200 M, × 63 and × 20), the selected areas of interest were marked using a permanent marker on the cover slips and identified under the Differential Interference Contrast (DIC) microscope (Leica, objectives Plan × 10/0.22 and × 20/0.30). The cell layer around the marked areas was then brushed off under the DIC microscope using a fine needle. The samples were carefully washed to remove oil and scraped cells and subjected to a standard SEM procedure as described above.

### Time-lapse microscopy

MCF7 cells were seeded in the culture flasks, which had a coordinate grid on the bottom to locate cells of interest. Cells were incubated in a humidified incubator supplied with 5% CO_2_ at 37 °C. After 15 hours, the cell-in-cell structures were selected and marked using the DIC microscope equipped with HI Plan × 20/0.30 objective. The images were taken every hour for 80 hours. In total, thirteen MCF7 entotic cells were traced in three independent experiments.

### Statistical analysis

The frequency of entotic cells was represented as a percentage (mean ± SD) of the total cells counted (n = 1,000 cells). Data were obtained in three independent experiments. Plotting and calculation of the standard deviation value were made using Microsoft Office Excel 2007 software. Data were analyzed using the Analysis of Variance (ANOVA) test. P values < 0.05 were considered significant.

## Electronic supplementary material

Supplementary materials

Movie 1

Movie 2
