# Supplementary material for: Consecutive entosis stages in human substrate-dependent cultured cells
Source: Sci Rep. 2017 Oct 2;7:12555. doi: 10.1038/s41598-017-12867-6 (PMC5624876; doi:10.1038/s41598-017-12867-6)
Supplement: Supplementary file 1 — Supplementary materials [file 41598_2017_12867_MOESM1_ESM.pdf]

Consecutive entosis stages in human substrate-dependent cultured cells

Authors: Anastasiia S. Garanina[1], Olga P. Kisurina-Evgenieva[1], Maria V. Erokhina[1], Elena A. Smirnova[1], Valentina M. Factor[2] & Galina E. Onishchenko[1]*

Authors affiliations: [1]Department of Cell Biology and Histology, Faculty of Biology, Lomonosov Moscow State University, Russia. [2]Laboratory of Molecular Pharmacology, Center for Cancer Research, National Cancer Institute, National Institutes of Health, Bethesda, MD, USA.

Correspondence: GE Onishchenko, 119234, Leninskiye Gory, Moscow, Russia. Tel. +7 (495) 939-45-67. E-mail: galina22@mail.ru

Supplementary materials includes:

1. **Supplementary figures**
2. **Supplementary figure legends**

Supplementary Fig. S1.

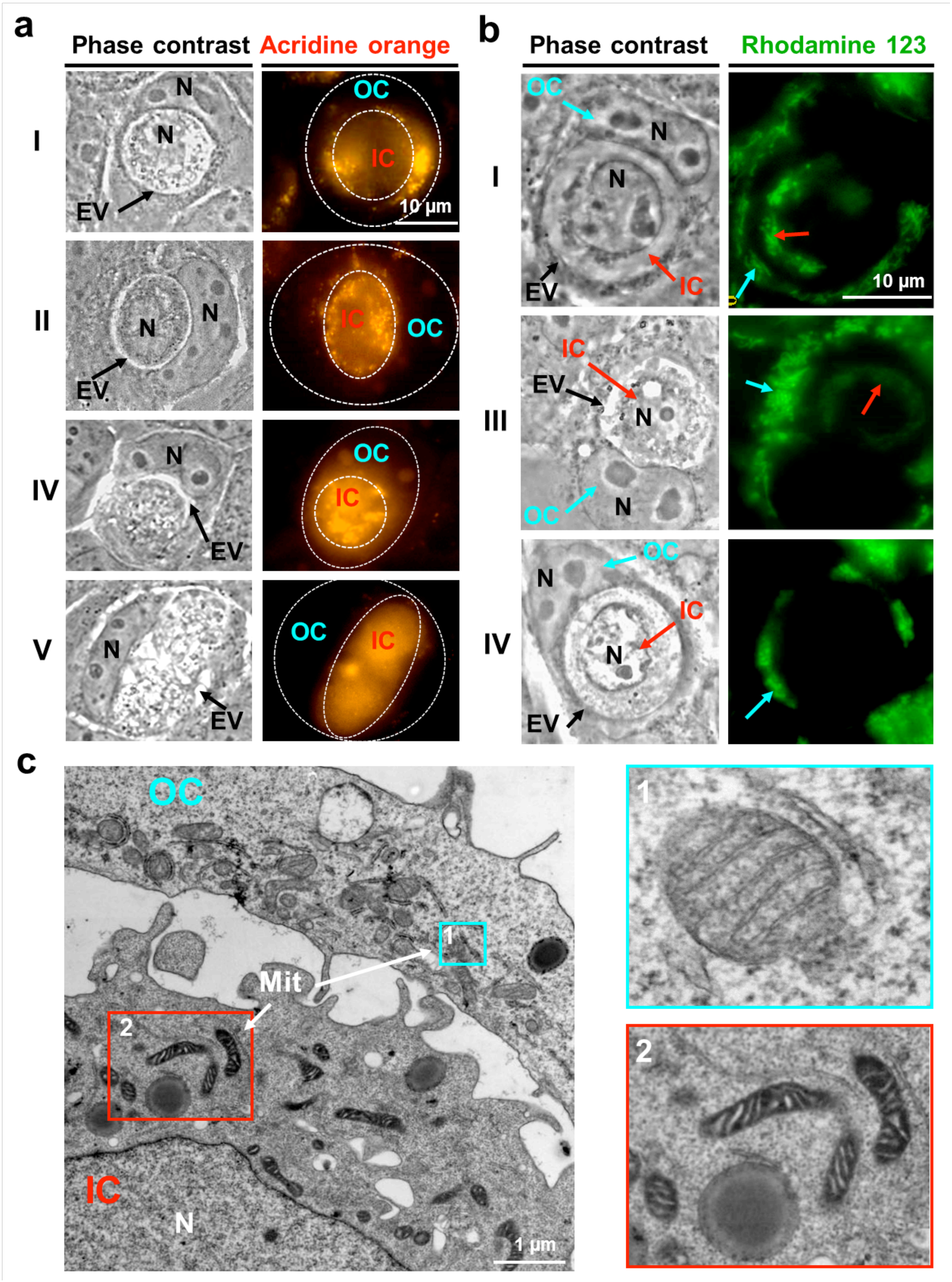

Supplementary Fig. S2.

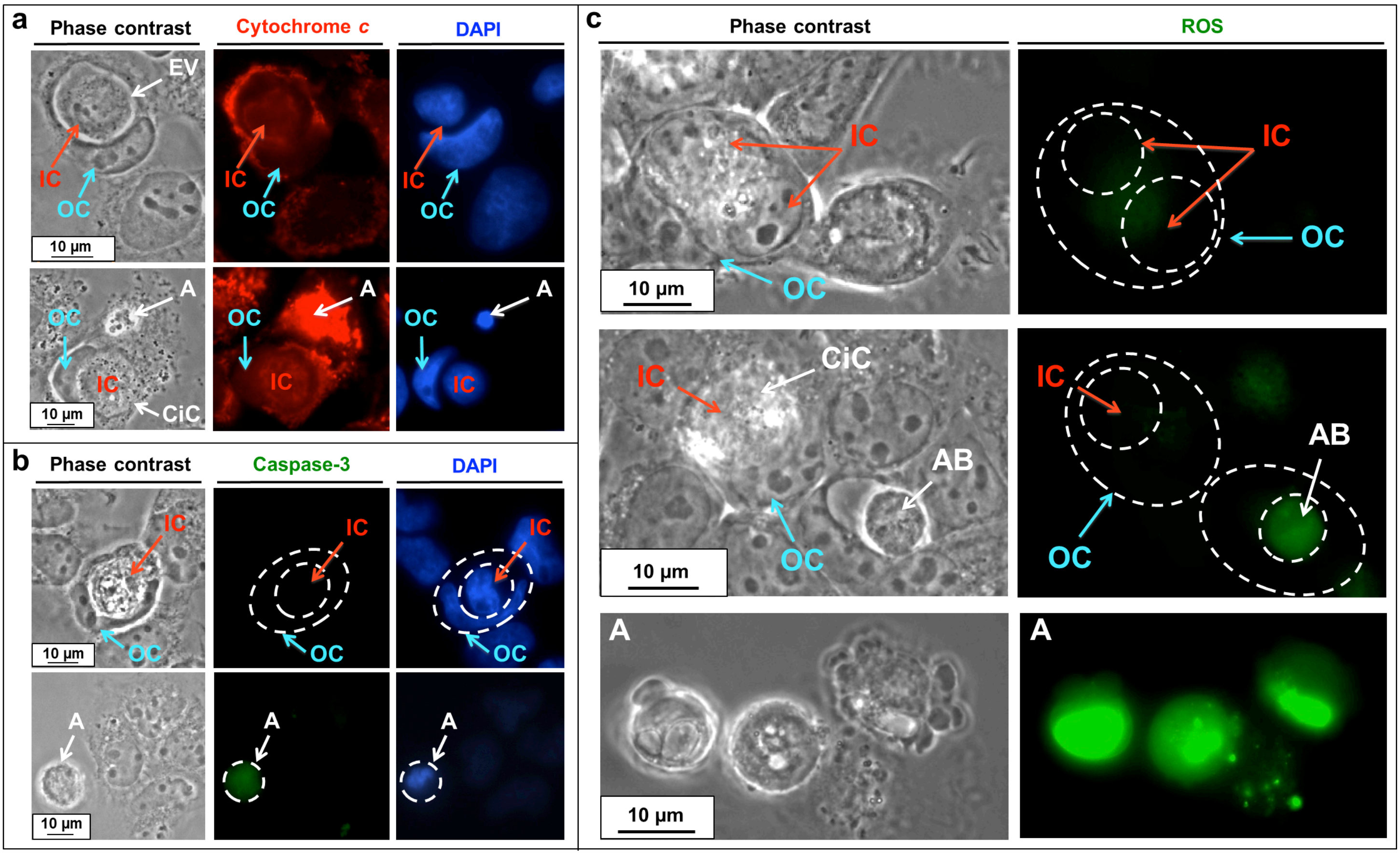

Supplementary Fig. S1. Changes in lysosome and mitochondrion compartments during entosis in A431 cell monolayer. (**a**) Localization of the acid vesicular compartment in the inner (IC) and outer (OC) cells at different entosis stages. Shown are representative phase-contrast and the corresponding fluorescence micrographs of entotic cells stained with acridine orange (orange). (**b**) Phase-contrast (left) and vital fluorescence (right) micrographs of IC and OC mitochondria at stages I, III and IV. A gradual decline of IC mitochondrial activity during entosis and redistribution of OC mitochondria are shown. Cells are stained with rhodamine 123. Red arrows, the mitochondria of the ICs; blue arrows, the mitochondria of the entotic cells. (**c**) Transmission electron micrograph of mitochondria in OC and IC (stage II). Panels 1 and 2 on the right are the magnified views of the boxed areas outlined in blue (1) and red (2). Morphological changes of IC mitochondria are shown. EV, entotic vacuole; Mit, mitochondrion; N, nucleus.

Supplementary Fig. S2. Entosis is different from apoptosis. **(a)** Representative phase-contrast (left) and fluorescence micrographs of entotic and apoptotic cells stained with anti-cytochrome *c* antibodies (middle) and DAPI (right). Note, that during entosis, there is no release of cytochrome *c* from inner cell (IC) mitochondria (top) characteristic for apoptosis (bottom). (**b**) Representative phase-contrast and fluorescent micrographs of entotic (top) and apoptotic (bottom) cells stained with anti-caspase-3 antibodies (middle) and DAPI (right). Caspase-3 is not activated during entosis (top) as compared to apoptosis (bottom). (**c**) Representative phase-contrast and fluorescence micrographs of entotic and apoptotic cells (A, bottom) stained with DCFH-DA, a marker of ROS (reactive oxygen species) accumulation. Increased ROS staining is found only in apoptotic body (AB) and apoptotic cells (A, bottom). A, apoptotic cells; AB, apoptotic body; CiC, cell-in-cell structure; EV, entotic vacuole; OC, outer cell; IC, inner cell.
